# Mitochondrial genome insights into the spatio-temporal distribution and genetic diversity of *Dendrobium hancockii* Rolfe (Orchidaceae)

**DOI:** 10.3389/fpls.2024.1469267

**Published:** 2024-10-22

**Authors:** Zhenyu Hou, Mengting Wang, Yu Jiang, Qingyun Xue, Wei Liu, Zhitao Niu, Xiaoyu Ding

**Affiliations:** ^1^ College of Life Science, Nanjing Normal University, Nanjing, China; ^2^ Jiangsu Provincial Engineering Research Center for Technical Industrialization for Dendrobiums, Nanjing, China; ^3^ Ningbo Key Laboratory of Agricultural Germplasm Resources Mining and Environmental Regulation, College of Science and Technology, Ningbo University, Cixi, China

**Keywords:** *Dendrobium hancockii*, mitochondrial genome, SNP, genetic diversity, nuclear single copy gene

## Abstract

**Introduction:**

With its distinctive evolutionary rate and inheritance patterns separate from the nuclear genome, mitochondrial genome analysis has become a prominent focus of current research. *Dendrobium hancockii* Rolfe, a species of orchid with both medicinal and horticultural value, will benefit from the application of the fully assembled and annotated mitochondrial genome. This will aid in elucidating its phylogenetic relationships, comparative genomics, and population genetic diversity.

**Methods:**

Based on sequencing results from Illumina combined with PacBio and Nanopore, the mitochondrial genome map of *D. hancockii* was constructed. Comparative analysis was conducted from the perspectives of phylogeny across multiple species, selection pressure on protein-coding genes, and homologous segments. The population diversity of *D. hancockii* was analyzed using single nucleotide polymorphism (SNP) data from the mitochondrial genome and single-copy nuclear genes.

**Results and discussion:**

This research constructed a circular mitochondrial map for *D. hancockii*, spanning 523,952 bp, containing 40 unique protein-coding genes, 37 transfer RNA genes, and 4 ribosomal RNA genes. Comparative analysis of mitochondrial genes from 26 land plants revealed a conserved gene cluster, "*rpl16-ccmFn-rps3-rps19*," particularly within the *Dendrobium* genus. The mitochondrial genome of *D. hancockii* exhibits a lower point mutation rate but significant structural variation. Analysis of 103 resequencing samples identified 19,101 SNP sites, dividing *D. hancockii* into two major groups with limited gene flow between them, as supported by population diversity, genetic structure analysis, principal component analysis, and phylogenetic trees. The geographical distribution and genetic differentiation of *D. hancockii* into two major groups suggest a clear phytogeographical division, likely driven by ancient geological or climatic events. The close alignment of mitochondrial data with nuclear gene data highlights the potential of the mitochondrial genome for future studies on genetic evolution in this species.

## 1 Introduction

The *Dendrobium hancockii*, known for its alkaloids (Bhardwaj et al., 2024), polysaccharides (Lai et al., 2024), and flavonoids (Meng et al., 2023) found in its stems, is commonly regarded as a herbal medicine (Dendrobii Herba) in East Asia. Additionally, belonging to the Orchidaceae family, the unique floral morphology of Dendrobium gives it potential as an ornamental plant (Zhang et al., 2022). As a species widely distributed throughout China (Pan et al., 2022), its range extends across the Sino-Himalayan Forest Subkingdom and the Sino-Japanese Floristic Region, showcasing its extensive environmental adaptability as both a horticultural and medicinal plant. However, samples from these different phytogeographic regions exhibit phenotypic variations, particularly in leaf shape, plant height, and floral morphology. Previous research has predominantly focused on the development and utilization of medicinal components, with limited studies on species evolution, phylogeny, and genetic diversity.

Compared to nuclear and plastid genomes, the mitochondrial genome has long been considered a key focus in evolutionary studies within genetic systems. This recognition stems from several characteristics, including its large size, complex and highly adaptable structure (Møller et al., 2021), varied nucleotide substitution rates (Drouin et al., 2008; Chevigny et al., 2020), and widespread horizontal gene transfer events (Waltz and Giegé, 2020). The exploration of mitochondrial genomes in green plants and their genetic reservoirs can be traced back to the pioneering work of Unseld (Unseld et al., 1997), which initially elucidated the mitochondrial genome of *Arabidopsis thaliana*. With advances in sequencing technologies and mitochondrial genome research, a vast structural diversity has now been revealed within plant mitochondrial genomes. This diversity is evident in single-ring mitochondrial genomes (Pinard et al., 2019) and multi-ring mitochondrial genomes (Lloyd et al., 2019). The variety of structures and differing evolutionary rates endow plant mitochondrial genomes with significant potential and applications in structural properties, homologous gene transfer, genetic architecture, and phylogeography.

Mitochondrial conformations often exhibit structural diversity mediated by repetitive sequences (Sun et al., 2022) or the insertion of foreign sequences (Choi et al., 2019). This structural diversity can occur even among closely related species within the same genus. Research on *Fragaria* (strawberries) indicates that such structural variability, rather than variability at gene loci, contributes to the rapid evolution of mitochondrial genomes (Fan et al., 2022). Furthermore, horizontal gene transfer among chloroplast, mitochondrial, and nuclear genomes, leading to derived fragments, also promotes species evolution (Kleine et al., 2009). The combination of foreign fragments with diverse structures, alongside recombinational changes, enables the mitochondrial genome to better adapt to evolving environments (Ma et al., 2022; Liu et al., 2013).

Furthermore, the dissimilarity in synonymous substitution rates between nuclear and plastid genomes also enhances the promise and value of mitochondrial-related systematic geography and biological evolution studies (Drouin et al., 2008). Utilizing molecular markers derived from the mitochondrial genome to investigate the origin, evolution, and dispersal patterns of mustard greens shows significant potential and prospects (You et al., 2022). However, research on the mitochondrial genome and its relevance in population evolution remains limited in most angiosperms, particularly in the case of orchids. To elucidate the mechanisms behind plant mitochondrial structural variations, replication, gene transfer events and genetic diversity, as well as their evolutionary history, a greater abundance of mitochondrial sequence data and comprehensive analyses are imperative.

Therefore, we initiated *de novo* sequencing to unravel the enigmatic mitochondrial genome of the rare *D. hancockii*, aiming to address the following critical inquiries: (a) furnish a comprehensive, high-quality mitochondrial genome assembly of *D. hancockii*, elucidating its structural attributes, sequence composition, and gene distribution within the species; (b) engage in comparative genomics analyses, leveraging our previously published *Dendrobium* mitochondrial genomes, to unearth potential molecular marker fragments and shed light on speciation events; (c) employ mitochondrial genome SNP data along with nuclear single-copy genes to conduct genetic structure investigations of *D. hancockii*, assessing the feasibility of employing plant mitochondrial genomes in genetic differentiation and systematic evolution.

## 2 Materials and methods

### 2.1 Materials and DNA extraction

The material used for sequencing the mitochondrial genome is the root tip tissue of tissue-cultured *D. hancockii* seedlings, sourced from the *D. hancockii* population in Pingbian, Yunnan. The remaining individual samples of the *D. hancockii* population are either plant or leaf samples collected by the research team from 2019 to 2021 from various provinces, including Yunnan, Guangxi, Shanxi, Shaanxi, Hubei, etc. All samples have been identified as *D. hancockii*. The leaves are preserved using silica gel desiccant and stored in a -80°C refrigerator. The plants are maintained in the cultivation room at the Institute of Plant Resources and Environment, College of Life Sciences, Nanjing Normal University.

Total DNA was extracted using 0.2 g of fresh plant tissue using the CATB method (Xu et al., 2012), followed by agarose gel electrophoresis to detect the quality of DNA; the concentration of DNA was measured by spectrophotometry (Preuten et al., 2010), and all samples were diluted to the same concentration. According to the *D. hancockii* resequencing data mapped to the *D.officinale* reference genome (Niu et al., 2021) using CLC Genomics Workbench 8.5.1 (CLC Biosciences, Aarhus, Denmark), 8 homologous genes were selected. Subsequently, 11 pairs of primers were designed for these genes or gene fragments using Primer Premier 5.0 (Singh et al., 1998), as listed in **Supplementary Table S1**. The PCR reaction followed a standard procedure: initial denaturation at 94°C for 5 min, 30 cycles (denaturation at 95°C for 15 s → annealing at Tm for 30 s → extension at 72°C for 30 s), and a final extension at 72°C for 5 min.

Mitochondria were isolated from 5 g of root tip tissue of *D. hancockii* using a modified method involving differential centrifugation and density gradient centrifugation (Gui et al., 2016). Mitochondrial DNA was extracted from purified mitochondria using a modified SDS method (Chen et al., 2011). Check the quality of mtDNA by agarose gel electrophoresis. DNA samples meeting the quality requirements (concentration ≥ 50 ng/μL, A260/280 = 1.8-2.0, A260/230 >1.7) were used for sequencing.

### 2.2 DNA sequencing, mitochondrial genome assembly and annotation

Sequence: Purified mtDNA of *D. hancockii* species was sequenced on Illumina Hiseq. Platform (Illumina, San Diego, CA, USA) generated 150 bp paired-end reads (with an insert size of 450 bp) and obtained approximately 4.2 Gb of raw data. Low-quality reads were trimmed using CLC Genomics Workbench 8.5.1 (CLC Biosciences, Aarhus, Denmark). In addition, the PacBio (Pacific Biosciences, Menlo Park, CA, USA) and Nanopore (Oxford Nanopore Biosciences, Cambridge, MA, USA) platforms generated approximately 43817 long reads from *D. hancockii*. Corresponding Illumina pair-end reads were used to correct erroneous bases with PacBio (nanopore) and indels were read using LoRDEC (kmer value = 19; abundance threshold = 3) (Salmela and Rivals, 2014).

Assembly: Firstly, the Illumina sequencing data was preliminarily assembled using ABySS v2.0.2 (http://www.bcgsc.ca/platform/bioinfo/software/abyss) (Jackman et al., 2017). Subsequently, the Nanopore third-generation data was aligned using blasR, and based on the alignment results, a correction and error correction were applied to the single-molecule sequencing data. The purpose of this step was to reduce errors such as single-base substitutions, insertions, and deletions in the long single-molecule sequences. Finally, the corrected single-molecule sequencing data was combined with the second-generation data for hybrid assembly using SPAdes-3.13.0 software (kmer values =33; phred-offset = 33) (Meleshko et al., 2019). High-coverage and long-assembled sequences were selected as candidate sequences, and the mitochondrial scaffold sequences were confirmed by aligning against the NT library. Clean reads were aligned back to the assembled mitochondrial scaffolds. Based on the paired-end and overlap relationships of the reads, local assembly and optimization were performed on the assembly results to determine the order and direction of the scaffolds. Subsequently, the GapCloser software (v1.12, http://soap.genomics.org.cn/soapdenovo.html) (Luo et al., 2012) was employed to perform gap filling on the assembly results, resulting in the final mitochondrial genome sequence.

Annotation: We employed a homology-based prediction method for gene prediction in the sample genome. Initially, the protein sequences of 36 angiosperm mitochondrial reference genomes (**Supplementary Table S2**) were rapidly aligned to the sample genome using BLAST+ 2.71 software with the parameter set as (e-value <1e−5). After filtering out poor alignment results and removing redundancies, manual correction of the sample genes’ integrity and exon/intron boundaries was performed to obtain a highly accurate set of conserved genes. Homology prediction and software such as tRNAscan-SE 2.0.4 (Schattner et al., 2005) were used to predict non-coding RNAs (ncRNAs) contained in the genome.

### 2.3 Chloroplast to mitochondrion DNA transfer and RNA editing analyses

Prediction of RNA editing sites in protein-coding genes using PREP-mt (Mower, 2009). The cutoff value was set as default. RE sites of double-copy genes were counted twice. To identify plastid-derived fragments, the mitogenome of *D. hancockii* was compared against the plastomes of *D. hancockii* (LC500592.1) (Hou et al., 2019) using BLAST v2.11.0+ (Camacho et al., 2009), with criteria set as a matching rate of ≥ 70%, E-value of ≤ 1e − 10, and length of ≥ 40 bp.

### 2.4 Analysis of repeated sequences

The Microsatellite identification tool facilitated the detection of simple sequence repeats (Beier et al., 2017) (https://webblast.ipk-gatersleben.de/misa/index.php). Tandem repeats with a repeat unit of more than 6p were identified utilizing Tandem Repeats Finder v4.09 software (Benson, 1999) (http://tandem.bu.edu/trf/trf.submit.options.html) employing default parameters. Direct and inverted repeats were identified using REPuter software (Kurtz et al., 2001) (https://bibiserv.cebitec.uni-bielefeld.de/reputer) with a minimal repeat size threshold set at 20 bp.

Co-linearity analysis employed two methods. To better illustrate the relationships among the Locally Collinear Blocks (LCBs) of the 26 mitochondria (**Supplementary Table S3**), we utilized the Mauve software (Darling et al., 2004) (http://darlinglab.org/mauve/mauve.html) for co-linearity analysis. For the identification of conserved gene clusters in the *Dendrobium* genus, we employed the MCScanX software (Wang et al., 2012) (https://github.com/wyp1125/MCScanX) for co-linearity analysis.

### 2.5 Analysis of codon usage bias and calculation of selective pressure

The MEGA X software (Kumar et al., 2018) was utilized to calculate the Relative Synonymous Codon Usage (RSCU) values of Protein-Coding Genes (PCGs) and their corresponding amino acid composition. Codon preferences were established through the implementation of Perl scripts. The non-synonymous (Ka) and synonymous (Ks) substitution rates were computed using DnaSP 5.10 (Librado and Rozas, 2009), considering a total of 13 shared PCGs among the mitogenomes of *D. hancockii* and 25 other land plants.

### 2.6 Read mapping and SNP calling

Before read mapping, Trimmomatic v.0.38 (Lohse et al., 2012) was employed to eliminate adapters and trim low-quality bases. Bases with a quality score below 20 were removed from the beginning or end of reads, and reads shorter than 36 bases after trimming were discarded. The quality of reads was evaluated using FastQC v.0.11.7 (https://www.bioinformatics.babraham.ac.uk/projects/fastqc/, last accessed October 23, 2019) both before and after trimming. The remaining read pairs were aligned to the complete mitochondrial genome of *D. hancockii* using BWA-MEM v.0.7.17 (parameter: mem -t 4, -k 32 -M) (Li, 2013) and subsequently sorted with SAMtools v.1.9 (parameter: r m d u p) (Li et al., 2009). The MarkDuplicates tool from Picard v.2.18.21 (http://broadinstitute.github.io/picard/, last accessed March 16, 2019) was then applied to identify and mark PCR duplicates, while realignment around indels was performed using GATK v.3.8.1 (DePristo et al., 2011). Ultimately, GATK v.3.8.1 HaplotypeCaller was utilized for per-sample variant detection, and the GenotypeGVCFs tool was employed for joint genotyping. Given that our dataset comprises individuals from multiple species, we utilized the “EMIT_ALL_SITES” flag to generate a “mutation sites” VCF, which includes both variant and nonvariant sites, during SNP calling and genotyping.

### 2.7 Phylogenetic analysis

Protein-coding sequences of 13 mitochondrial genes and complete mitochondrial genome that were shared by 26 species (include two outgroups, *Psilotum nudum* and *Funaria hygrometrica*), were used to construct a constraint phylogeny for rate comparisons. Sequence alignments for each gene were performed using MAFFT 7.0 (Katoh and Standley, 2013). The maximum likelihood (ML) tree was constructed using Phylogenies were inferred by RAxMLv8.0.0 (Stamatakis, 2014) with (GTR + G + I) model and 1000 bootstrap replicates, and *Dendrobium kingianum* was selected as an outgroup IQ-TREE v.1.6.12 (Nguyen et al., 2015) with non-parametric bootstrap from 1000 pseudoreplicates. The neighbor-joining (NJ) tree (Tamura et al., 2004) was constructed using Phylogenies were inferred by MEGA X (Kumar et al., 2018) with Kimura 2-parameter model and 1000 bootstrap replicates. The ML/NJ tree with bootstrap support values was visualized using FigTree v.1.4.4 (https://tree.bio.ed.ac.uk/software/figtree/).

### 2.8 Population genetic diversity and genetic structure analysis

We identified nuclear gene sequences using version DnaSP 5.10 (Librado and Rozas, 2009) to calculate nucleotide diversity (π). Calculate the genetic differentiation index *Fst* and gene flow *Nm* (Nm ≈ (1 - *Fst*)/4*Fst*) between populations. Use ArcGIS ver. 10.2 (http://desktop.arcgis.com) software was used to create geographic maps. Use PERMUT to calculate intra-population gene diversity (*H_S_*), genetic diversity (*H_T_*) across all populations, interpopulation differentiation (*G_ST_)*, and number of surrogate types (*N_ST_*). The last two indices (*G_ST_* and *N_ST_*) were analyzed by a permutation test with 1000 permutations. Analysis of molecular variance (AMOVA) using Arlequin ver 3.5.2.246 divided different levels of genetic variation, statistically significant determined by 1,000 permutations, squared deviations (SSDs), Harpending’s raggedness index (HRI), and corresponding *P* values were calculated by Arlequin ver.3.5.2.246.

### 2.9 Structure and PCA analysis

The model-based program STRUCTURE (Raj et al., 2014) was used to determine the genetic structure of five populations of *D. hancokkii* by using mtSNPs and nuclear gene single-copy fragment combinations data (Falush et al., 2003). We specified a burn-in of 500,000 and set the MCMC iteration number to 1,200,000. We assumed an admixture model and correlated allele frequencies, and no prior information on taxon identity was included; default values were applied for all other parameters. The number of groups (K) ranged from 1 to 10 with 10 independent runs. The most likely number of clusters was determined by computing the natural logarithm of the likelihood function, and the ΔK statistic was calculated using Structure Harvester (Earl and vonHoldt, 2012). Conversely, for Principal Component Analysis (PCA) on the pruned SNPs, we utilized the smartpca program from EIGENSOFT v.7.2.1 (Patterson et al., 2006).

## 3 Results

### 3.1 Mitogenome structure and gene content

**Figure 1:**
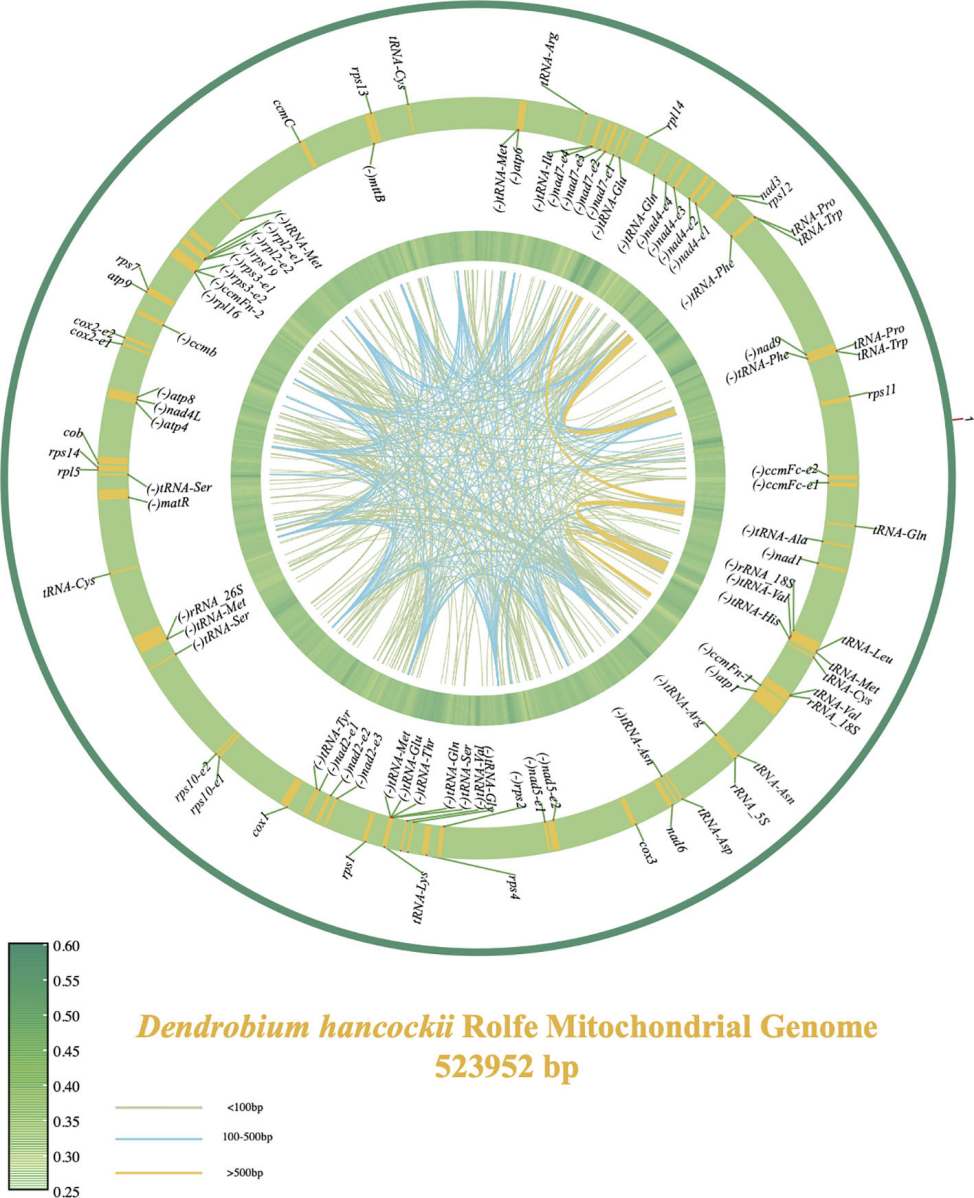
Mitochondrial genome maps of *D. hancockii*. The central region of the plot represents the dispersed repeat region, with colors indicating the fragment lengths—light green for segments under 100bp, blue for segments between 100bp and 500bp, and yellow for segments over 500bp. The green heatmap ring represents the GC content of the mitochondrial genome (with a sliding window of 1000 bp). The alternating yellow and green ring illustrates the distribution of mitochondrial genes, where yellow areas represent the coding sequence/exonic region of the gene, with the inner side indicating the reverse orientation and the outer side indicating the forward orientation.

The mitochondrial genome of tissue-cultured *D. hancockii* root tip cells was meticulously assembled into a single, large circular molecule, boasting a total length of 523,952 base pairs (bp) and an overall glycine cysteine content of 44.28% (Accession number: LC771085). Thorough annotation efforts resulted in the prediction and annotation of a total of 81 genes within the mitochondrial genome, including 40 protein-coding genes (PCGs), 4 ribosomal RNA (rRNA) genes, and 37 transfer RNA (tRNA) genes (**Figure 1**).

Of the 40 conserved PCGs present in all plant mitochondrial genomes, 17 genes encode vital components related to electron transport proteins and ATP synthase. This set includes nine subunits of complex I (*nad*1, 2, 3, 4, 5, 6, 7, 9, and 4L), one subunit of complex III (*cob*), three subunits of complex IV (*cox*1, 2, 3), and five subunits of complex V (*atp*1, 4, 6, 8, and 9). Furthermore, four genes were identified for large ribosomal proteins (*rpl*2, 5, 14, 16), eleven genes for small ribosomal proteins (*rps*1, 2, 3, 4, 7, 10, 11, 12, 13, 14, and 19), four genes for cytochrome c biogenesis (*ccmB*, *ccmC*, *ccmFn*, and *ccmFc*), and two genes for maturase and transporter functions (*matR* and *mttB*). The combined length of the 40 PCGs, ranging from 273 bp (*nad4L*) to 1,905 bp (*matR*), totaled 34,116 bp, accounting for 6.5% of the entire mitochondrial genome. According to the Relative Synonymous Codon Usage (RSCU) analysis presented in (**Supplementary Figure S1**), it is evident that leucine constitutes the predominant amino acid in the coding genes, accounting for 9.62% of the total codons with 1094 occurrences. Subsequently, serine is the second most prevalent, represented by 1071 codons, contributing to 9.42% of the total. Arginine follows with 894 codons, making up 7.86% of the overall composition, as revealed by the RSCU analysis.

Additionally, within the identified PCGs, six protein-coding genes (*ccmFc*, *cox2*, *rps3*, *nad5*, *rpl2*, *rps10*) contain one intron each. Furthermore, the *nad2* gene is characterized by the presence of two introns, while the *nad4* and *nad7* genes each contain three introns.

### 3.2 Analyses of repeat elements in *D. hancockii* mitogenome

Traditional analysis of mitochondrial repeats categorizes them into three types: simple repeats (SSR), tandem repeats, and scattered repeats. Through the utilization of an online Microsatellite Identification tool, we identified a total of 50 SSRs in the mitochondrial genome. These consist primarily of 46 mononucleotide repeat sequences, accounting for 92% of the total, alongside a small proportion (8%) of 4 dinucleotide repeat sequences (**Table 1**). Through the Tandem Repeats Finder tool, we identified 15 tandem repeats distributed evenly within the intergenic regions, with lengths ranging from 25 bp to 612 bp (**Supplementary Table S4**). Additionally, we employed the repetur software to quantify dispersed repeats within the mitochondrial genome. This analysis revealed a total of 437 dispersed repeats (larger than 20bp), as outlined in **Supplementary Table S5**. The combined length of these dispersed repeat sequences is 50,454 bp, accounting for 9.62% of the total length of the mitochondrial genome. Among these, repetitive segments with lengths less than 100 bp dominate, comprising 315 segments, accounting for 72.08% of the total segments. The lengths of 117 repetitive segments range from 100 bp to 500 bp, representing 26.77% of the total number of repeat segments, while the remaining 1.14% of segments have lengths exceeding 500 bp.

**Table 1:** Frequency of identifified SSR motifs in *D. hancockii* mitogenome.

Motif Type	Number of Repeats							Total	Proportion (%)
	6	7	8	9	10	11	12		
Monomer	0	0	0	0	32	11	3	46	92.00
Dimer	2	2	0	0	0	0	0	4	8.00
Trimer	0	0	0	0	0	0	0	0	0
Tetramer	0	0	0	0	0	0	0	0	0
Pentamer	0	0	0	0	0	0	0	0	0
Hexamer	0	0	0	0	0	0	0	0	0
Total	2	2	0	0	32	11	3	50	100

Monomer: Single nucleotide repeats; Dimer: Single nucleotide repeats.

### 3.3 Characterization of *D. hancockii* plastid genome transfer into the mitogenome

To identify chloroplast fragments within the mitochondrial genome, we utilized the mitochondrial genome as a reference and compared it with the published chloroplast genome (GenBank: LC500592.1). A total of 55 similar fragments were compared, ranging in length from 32 bp to 2434 bp, as outlined in **Supplementary Table S6**. These comparable sequences have a collective length of 31333 bp, constituting approximately 5.98% of the entire mitochondrial genome. Notably, a single plastid gene, *rpl14*, has been transferred and annotated in the mitochondrial genome of *D. hancockii*. We have identified nine intact tRNA genes of plastid origin in the mitochondrial genome. (*trnV-GAC*, *trnQ-UUG*, *trnL-CAA*, *trnM-CAU*, *trnV-GAC*, *trnQ-UUG*, *trnI-CAU*, *trnN-GUU*, *trnS-GCU*). Moreover, We validated the regional boundaries of mitochondrial genomes derived from partial plastids through PCR to ensure the reliability of assembly.

### 3.4 Comparative analysis of mitochondrial genomes

**Figure 2:**
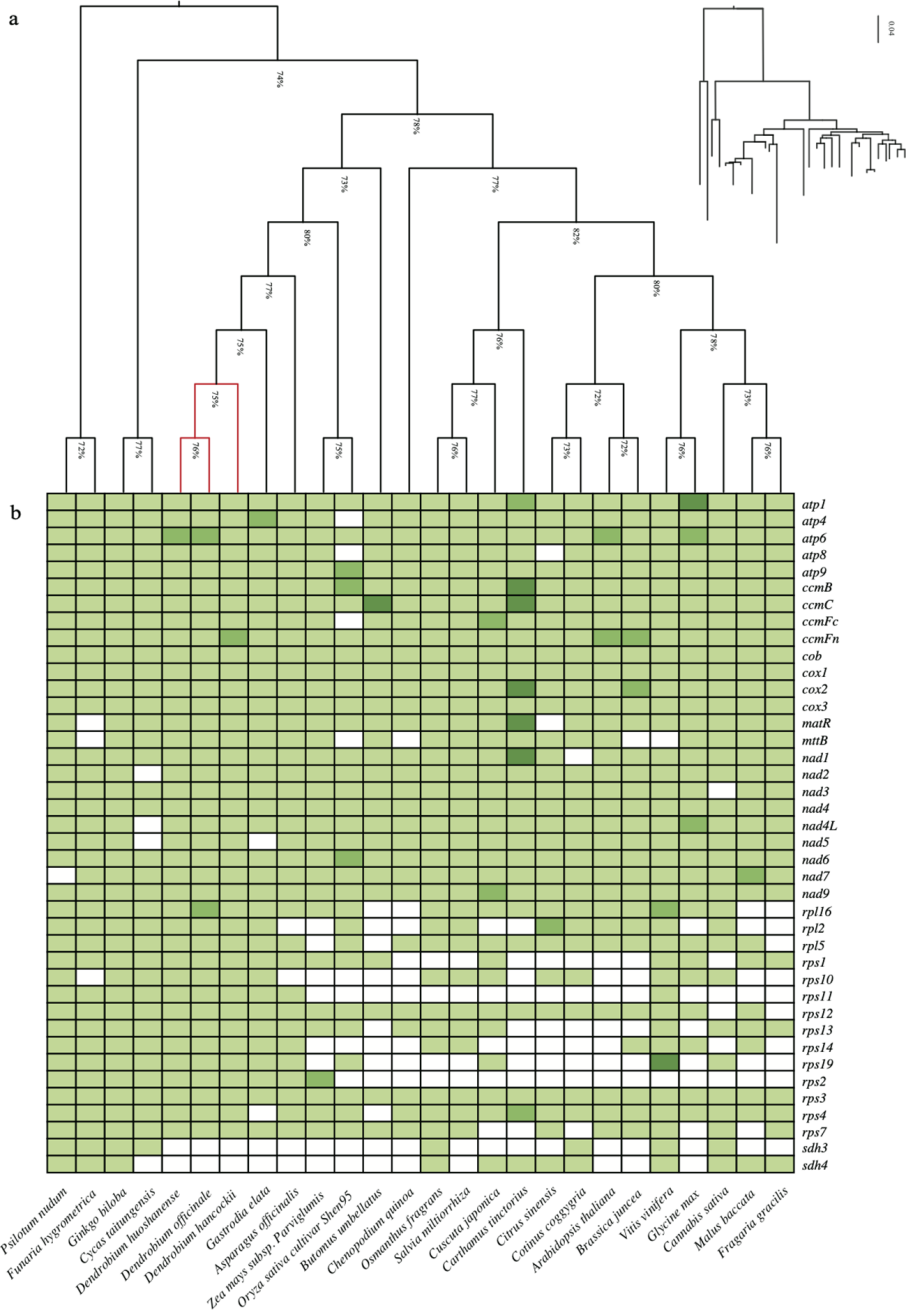
Comparison of the mitochondrial genes. **(A)** The NJ tree is constructed using the selected conserved mitochondrial genes set. **(B)** Distribution heatmap of mitochondrial genes across species. Colors indicate single‐copy (light green), double‐copy (green), triple-copy (dark green), and lost (white) genes.

Currently, we have conducted a comparative analysis of 26 plant mitochondrial genomes based on published data, encompassing a diverse collection of land plants including bryophytes, ferns, gymnosperms, and angiosperms (**Supplementary Table S3**). Among land plants, the key functional genes of mitochondria, particularly those associated with electron transport chains and oxidative phosphorylation (*atp*1, 6, 9, *ccmB*, *ccmC*, *ccmFc*, *ccmFn*), exhibit high conservation. On the contrary, the *sdh3* and *sdh4* genes, components of mitochondrial complex II, have undergone substantial loss (**Figure 2**). Differentiation in mitochondrial gene content among land plants predominantly arises from variations in ribosomal protein-related genes(*rps2*, *rps10*, *rps11*, *rpl2*, *rpl10*, *rpl14*).

**Figure 3:**
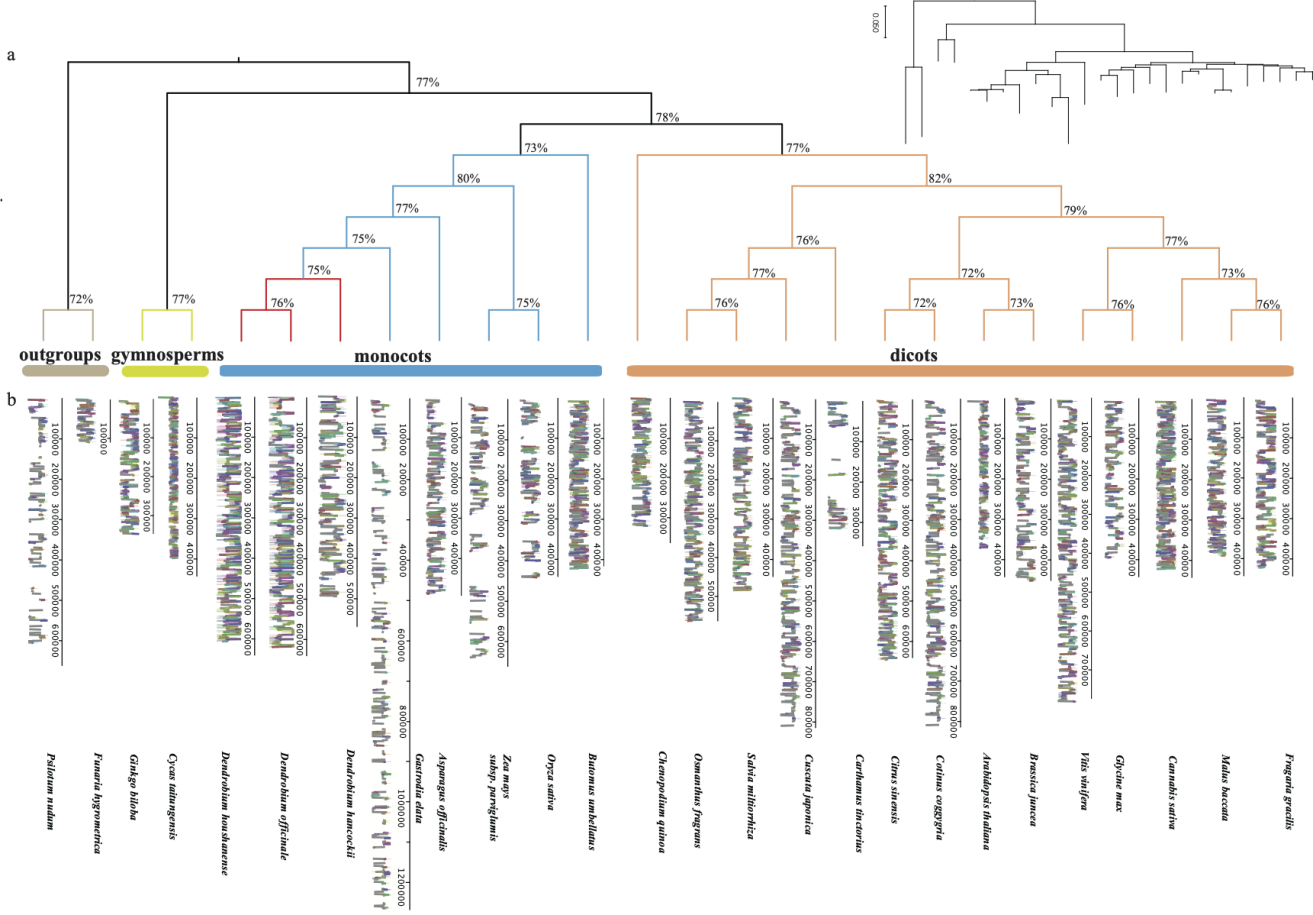
Comparison of mitochondrial genomes: **(A)** ML tree constructed with shared mitochondrial genome sequences, where the red region represents *Dendrobium* species; **(B)** Mauve comparison plot of the genomes of 26 land plants, with blocks of the same color indicating homology and the width of the blocks reflecting the similarity of genome sequences.

Moreover, we applied a selection process based on core mitochondrial genes to the aforementioned 26 land plants, resulting in the identification of 13 conserved genes(*atp*1, 6, 9, *ccmB*, *ccmC*, *ccmFn*, *cob*, *cox*1, *2*, *nad*4, 6, 9, rps*3*). These genes were then grouped together to construct a phylogenetic tree using the Neighbor-Joining method. The topology of the gene tree aligns with the known species tree, exhibiting a support rate exceeding 75%. Through phylogenetic analysis, we established the evolutionary relationships among the mitochondrial genomes of the 26 plants, representing the major taxa within land plants. Bryophytes and ferns were chosen as outgroups for the analysis. Based on the phylogenetic tree results, it distinctly indicates a parallel relationship between gymnosperms and angiosperms. Monocots and eudicots of angiosperms form a cluster, within which our primary research focus, *Dendrobium* spp., is grouped together with *Gastrodia elata* in the Orchidaceae branch. In addition to the Neighbor-Joining method (**Figure 2A**), we utilized the Maximum Likelihood method for phylogenetic tree analysis (**Figure 3A**). This method also classified *D. hancockii* as closely related to *D. officinale* and *D. huoshanense* within the Orchidaceae family of the Asparagales order. Moreover, phylogenetic trees constructed from tandem sequences of conserved genes robustly support the evolutionary relationships of plants, as established by the systematic taxonomy of the Angiosperm Phylogeny Group (APG IV).

In the field of mitochondrial genomics, as shown in **Figure 3B**, it is evident that the size of terrestrial plant mitochondria ranges from 100 kb to 1400 kb. Although significant variations in length have been observed in the orchid *Gastrodia elata*, MAUVE analysis indicates that most functional genes in the mitochondrial genomes of terrestrial plants are highly homologous, represented by similarly colored blocks in homologous regions. This results in a stable average genome size between 400 kb and 500 kb. However, mitochondrial genomes outside this range show significant differences in intergenic regions. Focusing on the eight orchid species (**Supplementary Figure S2**; **Supplementary Table S7**), both the number and length of homologous mitochondrial sequences increase, suggesting closer genetic relationships among these species. However, the connections between homologous regions reveal that the arrangement of these sequences differs, indicating extensive rearrangement events in orchid mitochondrial genomes. Notably, *D. officinale*, *D. hancockii*, and *D. huoshanense* show the closest relationships, with species from the *Dendrobium* genus exhibiting high levels of homology. Some homologous segments cluster into longer homologous gene clusters. We identified five larger gene clusters: *ccmFn-atp1*, *atp4-nad4L-atp8*, *rpl16-ccmFn-rps3-rps19*, *mttB-rps13*, and *nad3-rps12*. The specific locations of these fragments have been marked within the corresponding genomic regions (**Supplementary Figure S2**).

### 3.5 The substitution rates of mitochondrial PCGs

**Figure 4:**
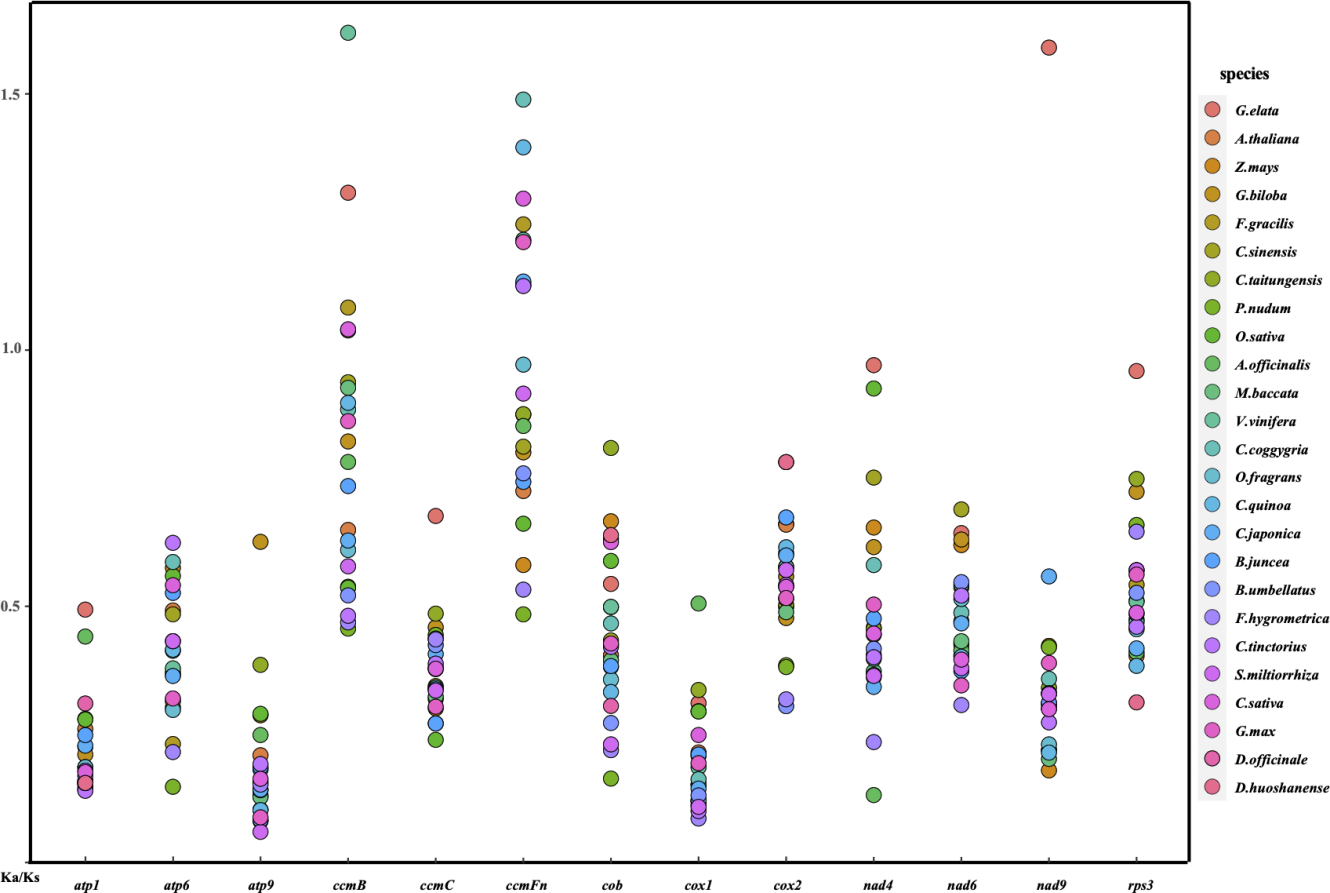
A dot plot of the Ka/Ks values of 13 protein-coding genes in mitogenomes of *D. hancockii* versus 25 species.

Utilizing a set of 13 conserved protein-coding genes, in conjunction with *D. hancockii* as a reference, a total of 26 terrestrial plant species were examined for the nonsynonymous to synonymous substitution ratio (Ka/Ks). After excluding cases where Ka/Ks was unavailable (0 or NA), it is evident from **Figure 4** and **Supplementary Table S8** that, in the majority of protein-coding genes (PCGs), the Ka/Ks values are significantly less than 1. This observation indicates that these genes have been predominantly subjected to purifying selection during the course of evolution. In contrast, within *ccmB*, when compared to *D. hancockii*, *G. elata*, *F.gracilis*, *C.taitungensis*, *V.vinifera*, and *C.sativa* exhibit Ka/Ks values exceeding 1, suggesting positive selection acting on this gene. Additionally, positive selection is observed in the *ccFmn* of *F.gracilis*, *M.baccata*, *V.vinifera*, *C.coggygria*, *C.quinoa*, *C.japonica*, *C.taitungensis*, *C.sativa*, *G.max*, as well as the *nad9* of *G. elata*. The Ka/Ks values for *nad4* and *rps3* in *G. elata* are close to 1, implying a trend towards neutral selection. On the other hand, certain genes such as *atp9*, *ccmC*, and *cox1* exhibit relatively small Ka/Ks values, indicating their conserved nature. Within the *Dendrobium* genus, Ka/Ks values exhibit lower values, with 12 out of 13 PCGs showing strong purifying selection. This result suggests a lower background nucleotide mutation rate among *Dendrobium* species.

### 3.6 Sequence variation and genetic diversity

The sequence composed of high-quality SNP (Single Nucleotide Polymorphism) sites of the entire mitochondrial genome was utilized to assess nucleotide diversity. SNP calling was performed on resequenced data obtained from 103 samples of *D. hancockii*, collected from 23 distinct wild populations (**Supplementary Table S9**). This analysis resulted in the identification of a total of 19,101 high-quality SNP sites. According to **Table 2**, the nucleotide polymorphism range for the 23 wild populations varied from 0.1933 (PB population) to 0.09579 (WC population). Based on geographical distribution and morphological characteristics, they were divided into two groups. Group1, consisting of 15 populations, exhibited a higher *P_i_* value (0.05660) compared to Group2, composed of 8 populations, with a *P_i_* value of 0.02387.To further validate the reliability of this data, a segment comprising a total of 12,927 bp, derived from 8 nuclear single-copy fragments, confirmed these results. The nucleotide diversity within this nuclear data ranged from 0.01035 (GJ population) to 0.01678 (BH population). Notably, the *P_i_* value for Group1 (0.01622) was higher than that of Group2 (0.01223).

**Table 2:** *P_i_* by population based on mitochondrial snp and nuclear genetic data.

Population	Nucleotide diversity of mt-data	Nucleotide diversity of nul-data
Group1	0.05660	0.01622
BH	0.04734	0.01678
BT	0.08070	0.01191
DDH	0.04503	0.01550
HX	0.01983	0.01521
KD	0.04931	0.01417
LP	0.03789	0.01552
NS	0.06591	0.01389
SNJ	0.07005	0.01524
SY	0.05308	0.01380
TB	0.02298	0.01492
WC	0.09579	0.01048
WD	0.05359	0.01492
WDS	0.04936	0.01368
WLS	0.04960	0.01365
YA	0.07594	0.01169
Group2	0.02387	0.01223
GJ	0.01518	0.01035
JS	0.03116	0.01276
PB	0.01933	0.01111
TC	0.02359	0.01092
TL	0.01439	0.01121
WM	0.02327	0.01173
XY	0.03900	0.01095
ZF	0.01997	0.01178

*P_i_*: Nucleotide diversity; Group1: BH (BaiHe), BT (BuTu), DDH (DaDuHe), HX (HuiXian), KD (KangDing), LP (LiangPing), NS (NingShan), SNJ (ShenNongJia), SY (ShanYang), TB (TianBao), WC (WenChuang), WD (WuDu), WDS (WuDangShan), WLS (WuLingShan), YA (YaAn); Group2: GJ (GeJiu), JS (JianShui), PB (PingBian), TC (TengChong), TL (TianLin), WM (WangMo), XY (XingYi), ZF (ZhenFeng).

In order to detect the genetic differences within the population, between the groups, within and between groups, the AMOVA analysis was performed using Arlequin software, and the results showed that the variation within the population (64.56%) was greater than the variation between the total population (35.44%), and the genetic differentiation value was *Φ_ST_* = 0.35440 (**Table 3**). According to the geographical distribution of *D. hancockii* was divided into two groups, the AMOVA analysis of the two groups was as follows: the among group genetic variation was (53.32%) greater than the within-populations genetic variation (45.57%) and the within-group genetic variation (1.11%), and the corresponding genetic differentiation values were *Φ_CT_* = 0.53320, *Φ_ST_* = 0.54425 and *Φ_SC_* = 0.02368. Based on the mitochondrial SNPs, the variation of *D.hancockii* came from within the populations, and the differences among groups had a greater impact on the variation of *D.hancockii*, and its influence had a greater impact on the variation of *D.hancockii* than the differences among populations and within the group. The same analysis was also conducted on nuclear data, revealing that whether considering all 23 populations (70.73%) or the two groups (56.72%), the results indicate that a greater amount of genetic variation occurs inter-population. In summary, the variation of *D.hancockii* mainly came from among-group and inter-population.

**Table 3:** AMOVA analysis of population based on nuclear genes and mitochondrial genome of *D. hancockii*.

Data Type	Source of variation	*df*	Sum of squares	Variance components	Percentage variation	Fixation indices
Mito-SNP	All regions					
	Among populations	22	30760.77	222.18667 Va	35.44%	
	Within populations	80	32380.017	404.75021 Vb	64.56%	*Φ_*_ST_*_* =0.35440
	Total	102	63140.786	626.93688		
	Two groups					
	Among groups	1	21338.973	473.52963 Va	53.32%	*Φ_CT_* = 0.53320
	Among populations within groups	21	9421.797	9.81807 Vb	1.11%	*Φ_SC_* = 0.02368
	Within populations	80	32380.017	404.75021 Vc	45.57%	*Φ_ST_* = 0.54425
	Total	102	63140.786	888.09791		
Nul-data	All regions					
	Among populations	22	5412.31	35.71594 Va	29.27%	
	Within populations	80	6905.35	86.31687 Vb	70.73%	*Φ_ST_* = 0.29267
	Total	102	12317.660	122.03282		
	Two groups					
	Among groups	1	2548.736	54.68597Va	35.93%	*Φ_CT_* = 0.35932
	Among populations within groups	21	2863.574	11.19037Vb	7.35%	*Φ_SC_* = 0.11476
	Within populations	80	6905.35	86.31688Vc	56.72%	*Φ_ST_* = 0.43285
	Total	102	12317.66	152.19322		

df, degree of freedom; Φ*_CT_*, genetic variation among groups; Φ*_SC_*, genetic variation among populations within groups; Φ*_ST_*, genetic variation within populations.

**Figure 5:**
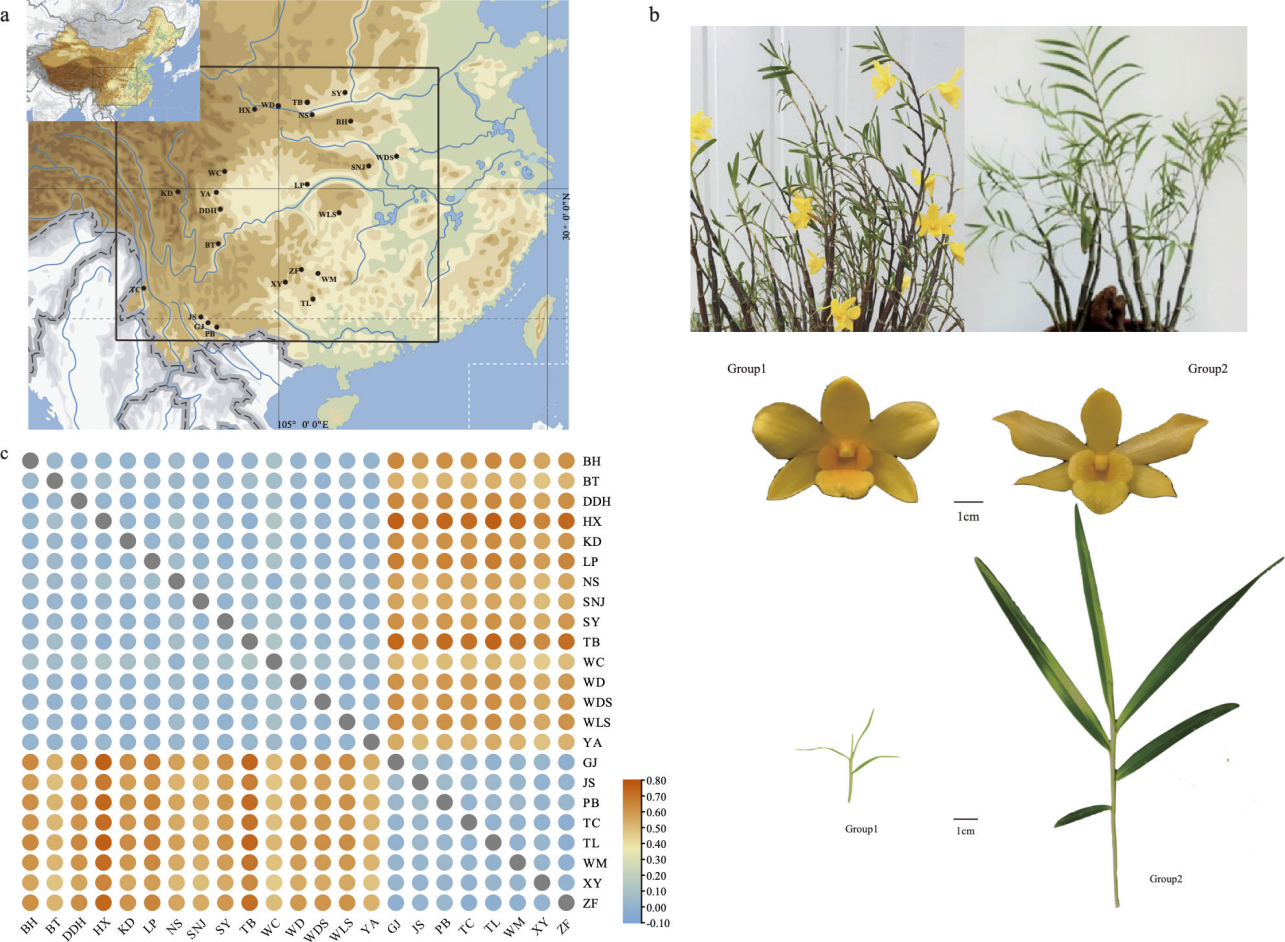
Phenotype, sampling locations, the genetic differentiation value of *D. hancockii* **(A)** Distribution of 23 wild populations; **(B)** Morphological phenotype information of *D. hancockii* in two groups; **(C)** The genetic differentiation value of 23 wild populations.

Through the calculation of genetic differentiation values and gene flow between populations, we gain insights into the extent of genetic variation among groups. The genetic differentiation value (*F_ST_*) was determined to be 0.42346, indicating a substantial degree of genetic differentiation between populations.This can also be observed from **Figure 5** and **Supplementary Table S10**. When comparing two populations, each falling within the two groups delineated based on geographical distribution and morphological differences, there is a noticeable difference in *F_ST_* among populations. The maximum *F_ST_* is observed between the HX population and TL population (*F_ST_* =0.76487). The gene flow (*N_m_=*0.0610) between the total populations was found to be less than 1, suggesting limited gene flow between these populations. Permut analysis utilizing Permut software revealed *G_ST_* and *N_ST_* values of 0.00068 and 0.43327, respectively, for the total population. The greater *N_ST_* value compared to *G_ST_* indicates the presence of a related geographical structure within the *D. hancockii* population, based on mitochondrial SNPs.

### 3.7 Genetic structure analysis

**Figure 6:**
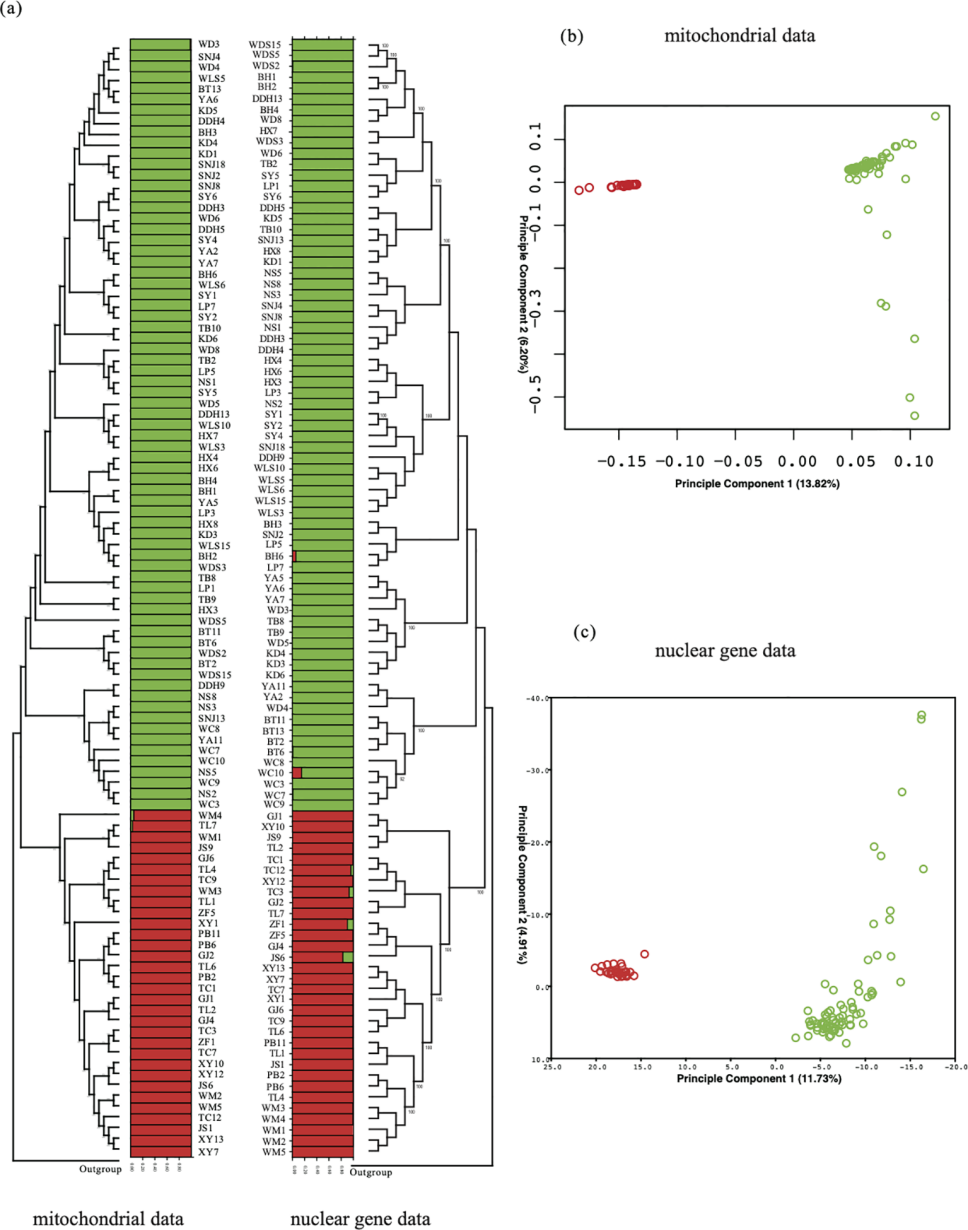
Gene structure analysis: ML tree, population structure, and PCA analysis were conducted using SNP data from the entire mitochondrial genome and nuclear single-copy gene data. **(A)** On the left are the ML tree and structure analysis based on mitochondrial data, while on the right are the results of the analysis based on the combination of nuclear single-copy genes. **(B, C)** PCA based on both mitochondrial and nuclear gene data.

The analysis of gene structure involved the fusion of mitochondrial genome SNPs and nuclear gene fragments data. To elucidate the genetic relationships among various populations and individuals, we employed several analytical approaches. According to the mitochondrial data in **Figure 6**, although the samples from various populations did not cluster into a single branch, they were effectively divided into two branches with high support. One branch exclusively consisted of samples from populations in the Group1 region, while the other branch comprised samples from the Group2 region. Similar outcomes were confirmed in nuclear data.

Despite some topological differences in the phylogenetic relationships between the two data types, both successfully separated the 103 samples into two branches, with relationships within each branch appearing more intricate, possibly due to higher similarity within each branch.In terms of PCA analysis, the mitochondrial data distinctly differentiated 32 samples from Group1 and 101 samples from Group2. The first two principal components, PC1 and PC2, accounted for 20.02% of the total genome covariance. The nuclear data analysis corroborated this result, where PC1 and PC2, representing 16.64% of the total genome covariance, also supported the separation of the entire sample set into two clusters, with 32 samples from Group1 and 101 samples from Group2 forming distinct clusters.

Furthermore, utilizing fastSTRUCTURE for structure analysis, the optimal K value was determined to be 2. When K=2 (**Figure 6**), the results effectively grouped all individuals into two distinct subgroups, aligning with the patterns observed in PCA and neighbor-joining trees. Specifically, minimal genetic admixture was observed in only two populations, WM and TL, in the Group2 region, indicating limited gene flow between the two subgroups. Similar results were found in the nuclear gene data’s structure analysis, where K=2 was the optimal value, dividing *Dendrobium* into two subgroups and detecting minimal gene flow among populations JC, TC, WC, and BH.

## 4 Discussion

### 4.1 Genomic architectural variation of *Dendrobium* mitochondria

In this study, we conducted the assembly of the mitochondrial genome of *D.hancockii*, spanning a length of 523,952 base pairs (bp). The mitogenome size is remarkably smaller than that of *D. officinale*, but larger than *D. gratiosissimum* (Wang et al., 2022). The assembly process utilized PacBio and Illumina sequence data, resulting in the identification of a monocyclic mitochondrial structure. The assembled genome consisted of a total of 40 coding genes and 41 non-coding RNAs, which were distributed across the genome. The GC content of the *D. hancockii* mitochondrial genome was found to be 44.28%, closely resembling that of related orchid species (42.9%-46.7%) (Ke et al., 2023). This observation suggests that the size and GC content of *Dendrobium* mitochondrial genomes has remained conserved throughout evolutionary processes. The acquisition of this result significantly contributes to the limited availability of mitochondrial genomic data in approximately 1500 *Dendrobium* species, thereby providing valuable molecular insights for future investigations.

In contrast to the conserved nature of mitochondrial sequence length and GC content in the *Dendrobium* genus, *D. hancockii* exhibits structural differences in its mitochondrial genome, featuring a singular main ring structure. This deviates from the previously reported multi-conformational patterns observed in two other *Dendrobium* species. Considering the potential involvement of extensive repetitive segments, possibly facilitating frequent and reversible homologous recombination events within the species, our findings contribute to the understanding of the emergence of multi-conformational phenomena in mitogenomes (Sloan et al., 2012). We identified the presence of large fragments (>500 bp) in five dispersed repeats, constituting 1.70% of the total genome length, capable of inducing conformational changes. Furthermore, our investigation of mitochondrial genomes in the *Dendrobium* genus, specifically *D. officinale* and *D. huoshanense*, utilizing PacBio and Illumina sequence data, disclosed a comparable number and proportion of large fragment repeats within the main ring structure of *D. hancockii* (Wang et al., 2022). This outcome suggests a potential role of substantial repetitive segments in contributing to conformational variations in the mitogenomic structure of the *Dendrobium* genus.

Reflecting on the study of structure of mitochondrial genomes, it is evident that research on plant mitochondrial genomes emerged later than studies on animal and bacterial mitochondria (Unseld et al., 1997). Consequently, scholars investigating plant mitochondrial genes have largely followed the established model. Initially, mitochondria predominantly exhibited a monocyclic structure, as observed in species such as *Camellia sinensis* (Li et al., 2023) and *Eucalyptus grandis* (Pinard et al., 2019). However, as research on the mitochondrial genomes of plants advanced, the concept of multichromosomal structures within mitochondrial genomes gained traction. This was first observed in the mitochondrial genome of *Cucumis sativus*, which was found to consist of three independent cyclic isomers (Alverson et al., 2011). Subsequently, the recognition of the diverse structural variability of mitochondria has gradually garnered acceptance among scholars. As demonstrated by our findings, the three species within the *Dendrobium* genus exhibit mitochondrial genomes with multiple structures. Variation in size and conformation within the mitochondrial genomes of related species is a common occurrence in plants (Sun et al., 2022). Consequently, we discourage the use of a specific structure to generalize the mitochondrial structure of *Dendrobium* plants, as both monocyclic and polycyclic structures possess inherent limitations. A monocyclic genomic map does not necessarily represent the absolute conformation of the mitochondrial genome within organisms, as alternative arrangements resulting from intramolecular or intermolecular recombination are plausible (Sloan, 2013). Such phenomena have been observed in *Photinia serratifolia* (Wang Y. et al., 2023). Similarly, polycyclic structures may arise due to changes in the mitochondrial genome prompted by environmental conditions. For instance, under stressful environments, plants may downregulate genes that typically inhibit recombination between short replicates of the mitochondrial genome. This mechanism may explain the extensive structural rearrangements and increased frequency of supercoiled DNA observed when plant tissues are cultured *in vitro* (Arrieta-Montiel and Mackenzie, 2011).

Consequently, we suggest the importance of understanding the diversity of mitochondrial genome conformations. Leveraging third-generation long-read DNA sequencing technology facilitates the high-quality assembly of plant mitochondrial genomes, facilitating more precise and sensitive detection of homologous recombination. This approach minimizes false positives introduced by PCR artifacts or limitations in read length inherent in next-generation sequencing methods (Schönegger et al., 2022). Through this approach, assembly discrepancies rather than true structural differences caused by sequencing issues are avoided. Hence, under the conditions of high sequencing depth, the assembly of mitochondrial genomes is poised to become a novel trend. Therefore, the utilization of high-depth sequencing techniques and next-generation sequencing technologies for the assembly of mitochondrial genomes is anticipated to emerge as a new trend. This approach aims to mitigate structural disparities caused by sequencing issues, offering rich and accurate data support for subsequent studies on mitochondrial genomes.

### 4.2 The mitochondrial genome mediating evolution through DNA fragment transfer.

The original mitochondrial genome is recognized for harboring a more comprehensive set of genes, reflecting functional evolution during its early evolution (Roger et al., 2017). However, owing to gene exchange between the nucleus and mitochondria, an increasing number of mitochondrial genes have translocated to the nuclear genome, leading to the depletion of genes from the mitochondrial genome itself (Mower, 2020). Similar phenomena are observed in organelle genomes as well (Kleine et al., 2009; Liu et al., 2013). In the mitochondrial genome of *D. hancockii*, this event occurred, with a total of 31,333 base pairs derived from the plastid, comprising approximately 5.98% of the total mitochondrial gene content. This proportion is consistent in plastid-originating mitochondria, ranging from 2 kb (0.5%) in *Vigna* genus plants to 113 kb (11.5%) in *Cucurbita* genus plants (Alverson et al., 2011). In the case of *D. hancockii*, the proportion closely aligns with that of *Apostasia shenzhenica* at 34 kb (5.12%) (Ke et al., 2023), in accordance with our expectations. The occurrence of similar evolutionary events in closely related species may potentially manifest in horizontal gene transfer at the genetic level. However, species-specific horizontal gene transfer events may occur. For example, the plastid-derived *rpl14* has been identified in the mitochondrial genome of *D. hancockii*, which is not observed in other species within the same genus (Wang et al., 2022). The horizontal gene transfer of *rpl14* from plastids to mitochondria may have taken place after the divergence of *D. hancockii* species. Furthermore, the occurrence of a similar event in the mitochondrial genome of *Vitis vinifera* suggests a multi-origin nature of horizontal gene transfer of *rpl14*.

It is crucial to note that a considerable proportion of migratory sequences have experienced degradation in their integrity over the course of evolution. Consequently, a limited number of genes have been discovered in the mitochondrial genome, the majority of which are tRNA genes. The findings indicate that tRNA genes exhibit a higher degree of conservation within the mitochondrial genome compared to the migratory patterns observed in protein-coding genes, emphasizing their crucial role within mitochondria. According to the analysis results, *D. hancockii* harbors nine tRNA genes derived from plastids, among which *trnN* and *trnM* exhibit relative conservation in terrestrial plants (Liao et al., 2018). This implies that these two tRNA genes may have undergone differentiation before the Orchidaceae divergence. Additionally, research indicates the loss of *trnV* in angiosperms, although it is present in certain dicotyledonous plants (Cheng et al., 2021). However, our study identifies the presence of *trnV*, suggesting frequent exchanges between mitochondrial and plastid genomes. Furthermore, the identification of *trnV* implies instances of gene loss and subsequent regain within the mitochondrial and plastid genomes, indicating dynamic evolutionary processes at play.

As widely acknowledged, a distinctive feature of plant mitochondrial genome evolution is the frequent insertion of foreign DNA or the horizontal transfer of mitochondrial DNA, including the transfer of its own plastid and nuclear DNA (Mower et al., 2012; Rose, 2021). Due to the unpublished status of the complete genome of *D. hancockii*, we are currently unable to determine whether there is any transfer of nuclear genes in the mitochondrial genome. However, based on the findings from published genome studies of *G. elata* (Yuan et al., 2018) and *D. officinale* (Niu et al., 2021), it is evident that transfer events between nuclear, mitochondrial, and chloroplast genomes occur within the orchid family.

### 4.3 Comparative analysis of mitochondrial genomes in 26 land plant species

To unravel the evolutionary history encoded in the mitochondrial genome, a comparative analysis of mitochondrial genomes was conducted among 26 green plants, including *D. hancockii*. Throughout the course of mitochondrial evolution in land plants, numerous instances of replication, loss, and rearrangement events have been identified. Despite these evolutionary dynamics, a set of highly conserved genes essential for maintaining mitochondrial functionality persists throughout (Karakaidos and Rampias, 2020). Thirteen conserved protein-coding genes (*atp*1, 6, 9, *ccmB*, *ccmC*, *ccmFn*, *cob*, *cox*1, 2, *nad*4, 6, 9, *rps3*) were identified from mitochondrial genome datasets of mosses (*Funaria hygrometrica*), ferns (*Psilotum nudum*), gymnosperms (*Ginkgo biloba*, *Cycas revoluta*), and angiosperms (including parasitic plants). According to Sloan DB (Sloan et al., 2008, Pfeifer et al., 2019), the majority of these PCGs are associated with the mitochondrial proton-translocating ATP synthase complex, cytochrome c biogenesis, mature enzyme functions, and transport membrane proteins. These results indicate that conserved mitochondrial genes serve indispensable and irreplaceable functions. Therefore, in the non-synonymous/synonymous substitution rate of conservative PCGs, the majority of Ka/Ks ratios are less than 1. However, genes *ccmb* and *ccmFn*, associated with cytochrome c, exhibit a significantly higher frequency of positive selection, implying that mutations in these genes may bring about adaptive functional changes. Moreover, these changes are more advantageous for survival. Thus, this phenomenon has been observed in various mitochondrial genes across many plant species, such as in *Suaeda glauca* (Cheng et al., 2021) and *Vitex rotundifolia* (Yu et al., 2022). Additionally, these PCGs can be leveraged to infer evolutionary relationships among species through phylogenetic analysis, with the resulting topology aligning with the mainstream APG IV system (APG group, 2016), underscoring the significant potential of plant mitochondrial genes in studying species evolution.

Concerning gene loss, intriguing results suggest that mosses (37 PCGs), ferns (39 PCGs), and even gymnosperms (40/37 PCGs) exhibit a greater diversity of mitochondrial genes, hinting at more frequent mitochondrial recombination events occurring after the evolution of angiosperms (Kan et al., 2021). At the species level within the family, higher levels of polymorphism are observed in the *rps* and *sdh* genes, as depicted by the gene cluster in **Figure 3**. This variation can be attributed to the deletion of mitochondrial genes resulting from the transfer of nuclear DNA and plastid DNA (Kleine et al., 2009). Further research into the comparative mitochondrial genomics of orchids has revealed varying degrees of gene rearrangement or loss (**Supplementary Figure S2**; **Supplementary Table S7**), which may be related to their adaptability to different environments. For example, among the eight orchid species, Gastrodia not only shows significant differences in mitochondrial structure but has also lost the relatively conserved genes *rps4* and *nad5*, possibly due to its mycoheterotrophic lifestyle. In orchids, closer phylogenetic relationships correspond to an increased number and length of homologous segments, which aligns with the principles of genetic evolution. Species within the same genus are more likely to retain a greater number of homologous segments during frequent structural changes. Through comparative analysis of the sequenced mitochondrial genomes of three species within the *Dendrobium* genus, we have not only revealed abundant homologous segments but also identified five conserved gene cluster fragments. This suggests that gene clusters may exhibit higher structural and sequence conservation. For instance, the identified “*rpl16*-*ccmFn*-*rps3*-*rps19*” gene cluster in this study displays significant similarity to the highly conserved “*rps19*-*rps3*-*rpl16*” gene cluster found in the fern *Adiantum capillus-veneris* (Bonavita and Regina, 2016). Therefore, we suggest that within the *Dendrobium* genus, mitochondrial diversity primarily exists in structural differences, manifested through variations such as repeat sequences, rearrangements, inversions, and other conformational changes. This pattern of rapid structural changes alongside slow sequence variation in the nucleotide background aligns with the mitochondrial evolution pattern observed in *Fragaria* species (Fan et al., 2022), and is consistent with research on *Paphiopedilum micranthum* within the Orchidaceae family (Yang et al., 2023).

### 4.4 The potential application of mitochondria in the analysis of genetic differentiation patterns

Firstly, the phylogeographic structure of *D. hancockii* and the major variations originating from among populations were confirmed. Analysis based on nuclear single-copy gene fragment data indicates that the *N_st_* value, reflecting the inter-population genetic differentiation coefficient, surpasses the *G_st_* value (*G_st_* = 0.00068, *N_st_* = 0.43327). This suggests the presence of a phylogeographic structure within *D. hancockii*, wherein populations that are closer geographically exhibit a closer genetic relationship. The majority of the genetic variation (64.56%) was found within populations, surpassing the among populations (35.44%). This finding aligns with results from related species, such as *D. officinale* (Hou et al., 2017) and *D. moniliforme* (Ye M. et al., 2017), where differentiation among wild populations is lower than within populations. The high differentiation observed among wild populations of *D. hancockii* suggests that gene flow between populations is limited compared to other *Dendrobium* species. Although there are no significant differences in seed and pollen dispersal among species within the same genus, the wide distribution of *D. hancockii* populations likely contributes to this phenomenon. Geographic isolation and the discontinuous distribution of populations also hinder gene flow (Pfeifer et al., 2019). Our analysis of gene flow confirms this assumption, as indicated by the value of nDNA: 0 < *N_m_* (0.06103) < 1, suggesting that overall, only some subpopulations of *D. hancockii* exhibit strong gene flow, which aligns with the results obtained from the phylogenetic analysis.

The results of phylogenetic reconstruction indicate that both mitochondrial and nuclear data support the division of the 103 *D. hancockii* samples into two groups. The population genetic structure analysis and PCA analysis (**Figure 6**) also support the viewpoint of dividing this species into two major lineages. The restricted gene flow between these lineages also implies that *D. hancockii* may have undergone outward expansion from two geographically distant refugia. By considering the influence of geological activity and glaciation on species expansion (Qiu et al., 2011), we suggest that the main factor contributing to the current phylogeographic pattern in *D. hancockii* is an adaptive response to ancient geological and climatic changes. Based on existing distribution information, we find that the division of wild *D. hancockii* populations into two major groups aligns with the “Tanaka-Kaiyong Line” division in the plant district flora (Ye J. et al., 2017). This alignment lends support to the phylogeographic division of *D. hancockii* and the boundary between the Sino-Himalayan forest subkingdom and Sino-Japanese forest subkingdom. This phenomenon has also been observed in other species, including *Sophora davidii* (Fan et al., 2013), *Tacca chantrieri* (Zhao and Zhang, 2015), *Leucomeris decora*, and *Nouelia insignis* (Zhao and Gong, 2015).

The integration of mitochondrial genome analysis with studies on phylogeography and genetic structures in animals is a well-established practice, as demonstrated in research on scorpions (Moreno-Carmona et al., 2023) and *Parasa sinica* (Zhang et al., 2023). This study marks the inaugural application of comprehensive mitochondrial genome SNP analysis for assessing genetic diversity and population structures in wild populations of *D. hancockii*. Analyses, encompassing genetic diversity, population genetic structure, and PCA, align closely with nuclear gene data, suggesting the potential utility of the mitochondrial genome in elucidating genetic differentiation patterns. Compared to nuclear gene data analysis, the results of mitochondrial analysis have confirmed its potential for application at the population level. However, it is undeniable that the use of mitochondrial genomes at the population level in species with narrow habitats and single distributions may present risks. Beyond the limitation of maternal inheritance in organellar genomes (Camus et al., 2022), the lower substitution rate of mitochondrial genomes (Drouin et al., 2008) may pose challenges when applied to endangered species with low diversity. However, due to the trans-regional distribution of *D. hancockii*, the impeded gene flow between populations caused by the pollen structure of Orchidaceae, and the climatic and geographic differences between northern and southern populations, the ‘good performance’ of the mitochondrial genome in *D. hancockii* may be revised by species-specific advantages. Therefore, the performance of the mitochondrial genome in population structure and genetic diversity needs further supplementary application across more species.

At the same time, the mitochondrial genome also has its unique advantages. Considering that certain heterotrophic parasitic plants, such as *G. elata*, have undergone the loss of photosynthetic genes (Xu et al., 2021), the mitochondrial genome emerges as a preferable choice for specific analyses. Studies reveal discrepancies in the phylogenetic relationships between plastid and mitotic genomes in *Aldrovanda vesiculosa* (Štorchová et al., 2023) and *Dendrobium* plants (Wang M. et al., 2023), suggesting unique applications and directions for different organelle genomes. Even within the *Potentilla* genus, the mitochondrial genome exhibits superior capabilities in constructing phylogenetic relationships compared to the chloroplast genome (Xue et al., 2023).

Current studies focused on plant phylogeography, genetic structure, and phylogenetics predominantly employ research methods that combine nuclear genes with organelle genes (Morris and Shaw, 2018). This approach has become mainstream, providing a more accurate reflection of incomplete lineage sorting (Steenwyk et al., 2023) or hybridization events (Fleming et al., 2023) during species formation, based on inconsistencies between nuclear and cytoplasmic genetic materials.The advent of mitochondrial genome analysis will enhance our exploration of plant genetic diversity, population structure, phylogeography, and systematics from a distinct perspective.

## 5 Conclusions

In this study, a comprehensive investigation was conducted on the rare plant *D. hancockii* through the application of second and third-generation sequencing methods. This approach allowed for *de novo* splicing assembly annotation, resulting in the successful acquisition of a circular and complete mitochondrial genome. The assembled genome revealed a cyclic structure and provided crucial information on 40 coding proteins, along with 37 tRNA and 4 rRNA genes. These findings significantly contribute to the enrichment of mitochondrial data within the *Dendrobium* species. Moreover, the study included a comparative analysis of the mitochondrial genome, shedding light on its evolutionary position and potential evolutionary events. The study also conducted comparative analyses of the mitochondrial genome, revealing its evolutionary position and potential evolutionary events. During the evolution of the *Dendrobium* genus, the mitochondrial structure demonstrates a pattern of variability while the sequence genes remain relatively conserved. Subsequently, a comprehensive population genetic structure analysis was conducted, utilizing the single nucleotide polymorphism sites within the mitochondrial group of 103 wild individuals. This data, combined with genetic structure analysis of nuclear single-copy gene sequences, led to the classification of *D. hancockii* from 23 distinct populations into two distinct subgroups. Furthermore, the study inferred that the genetic differentiation pattern of *D. hancockii* may be attributed to ancient geological and climatic factors This elucidates the potential of the mitochondrial genome in genealogical geography and contributes to a comprehensive understanding of the species’ evolutionary history.

## Data Availability

The datasets presented in this study can be found in online repositories. The data that support the findings of this study are openly available in the Science Data Bank at https://doi.org/10.57760/sciencedb.15636 & https://doi.org/10.57760/sciencedb.j00143.00099. The assembled mitochondrial genome sequences were submitted to the DDBJ with accession numbers: LC771085.
