# Supplementary material for: Mitochondrial genome insights into the spatio-temporal distribution and genetic diversity of Dendrobium hancockii Rolfe (Orchidaceae)
Source: Front Plant Sci. 2024 Oct 22;15:1469267. doi: 10.3389/fpls.2024.1469267 (PMC11535511; doi:10.3389/fpls.2024.1469267)
Supplement: Supplementary file 1 [file DataSheet1.zip › Supplementary Materials/Table S9 Sampling locations and sample numbers.docx]

Table S9 Sampling locations and sample numbers of *Dendrobium hancockii* populations

| Region Population code | Location | Latitude(N)  Longitude(E) | Number |  |
| --- | --- | --- | --- | --- |
| Group1 |  |  |  |  |
| BH | Baihe  Shaanxi province | 32°49'17.9"N 109°55'11.6"E | 5 |  |
| BT | Butuo  Sichuan province | 27°40'04.8"N 102°48'08.8"E | 4 |  |
| DDH | Daduhe  Sichuan province | 29°14'41.9"N 103°01'49.7"E | 5 |  |
| HX | Huixian  Gansu province | 33°50'57.3"N 106°01'21.3"E | 5 |  |
| KD | Kangding  Sichuan province | 29°37'33.5"N 101°43'05.7"E | 5 |  |
| LP | Liangping  Chongqing city | 30°36'42.9"N 107°31'19.5"E | 4 |  |
| NS | Ningshan  Shaanxi province | 33°45'39.6"N 108°32'34.3"E | 5 |  |
| SNJ | Shennongjia  Hubei province | 31°28'27.9"N 110°06'39.2"E | 5 |  |
| SY | Shanyang  Shaanxi province | 33°23'51.4"N 109°57'55.0"E | 5 |  |
| TB | Taibai  Shaanxi province | 33°55'24.2"N 107°16'04.7"E | 4 |  |
| WC | Wenchuan  Sichuan province | 31°05'40.4"N 103°17'44.8"E | 5 |  |
| WD | Wudu  Gansu province | 33°03'24.1"N 105°09'51.3"E | 5 |  |
| WDS | Wudangshan  Hubei province | 32°28'41.3"N 111°05'22.0"E | 4 |  |
| WLS | Wulingshan  Guizhou province | 27°54'49.8"N 108°41'09.2"E | 5 |  |
| YA | Yaan  Sichuan province | 30°02'25.8"N 103°00'23.5"E | 5 |  |
| Group2 |  |  |  |  |
| GJ | Gejiu  Yunnan province | 23°18'46.0"N 103°05'14.6"E | 4 |  |
| JS | Jianshui  Yunnan province | 23°33'43.5"N 102°42'38.8"E | 3 |  |
| PB | Pingbian  Yunnan province | 23°07'30.7"N 103°32'44.4"E | 3 |  |
| TC | Tengchong  Yunnan province | 25°01'25.0"N 98°33'03.1"E | 5 |  |
| TL | Tianlin  Guangxi province | 24°20'12.3"N 105°51'43.5"E | 5 |  |
| WM | Wangmo  Guizhou province | 25°08'49.5"N 106°04'51.6"E | 5 |  |
| XY | Xingyi  Guizhou province | 24°55'16.1"N 104°53'43.9"E | 5 |  |
| ZF | Zhenfeng  Guizhou Province | 25°23'25.5"N 105°34'57.2"E | 2 |  |
|  |  |  |  |  |
|  |  |  |  |  |
