# Supplementary material for: Mitochondrial genome insights into the spatio-temporal distribution and genetic diversity of Dendrobium hancockii Rolfe (Orchidaceae)
Source: Front Plant Sci. 2024 Oct 22;15:1469267. doi: 10.3389/fpls.2024.1469267 (PMC11535511; doi:10.3389/fpls.2024.1469267)
Supplement: Supplementary file 1 [file DataSheet1.zip › Supplementary Materials/Figure_S1.docx]

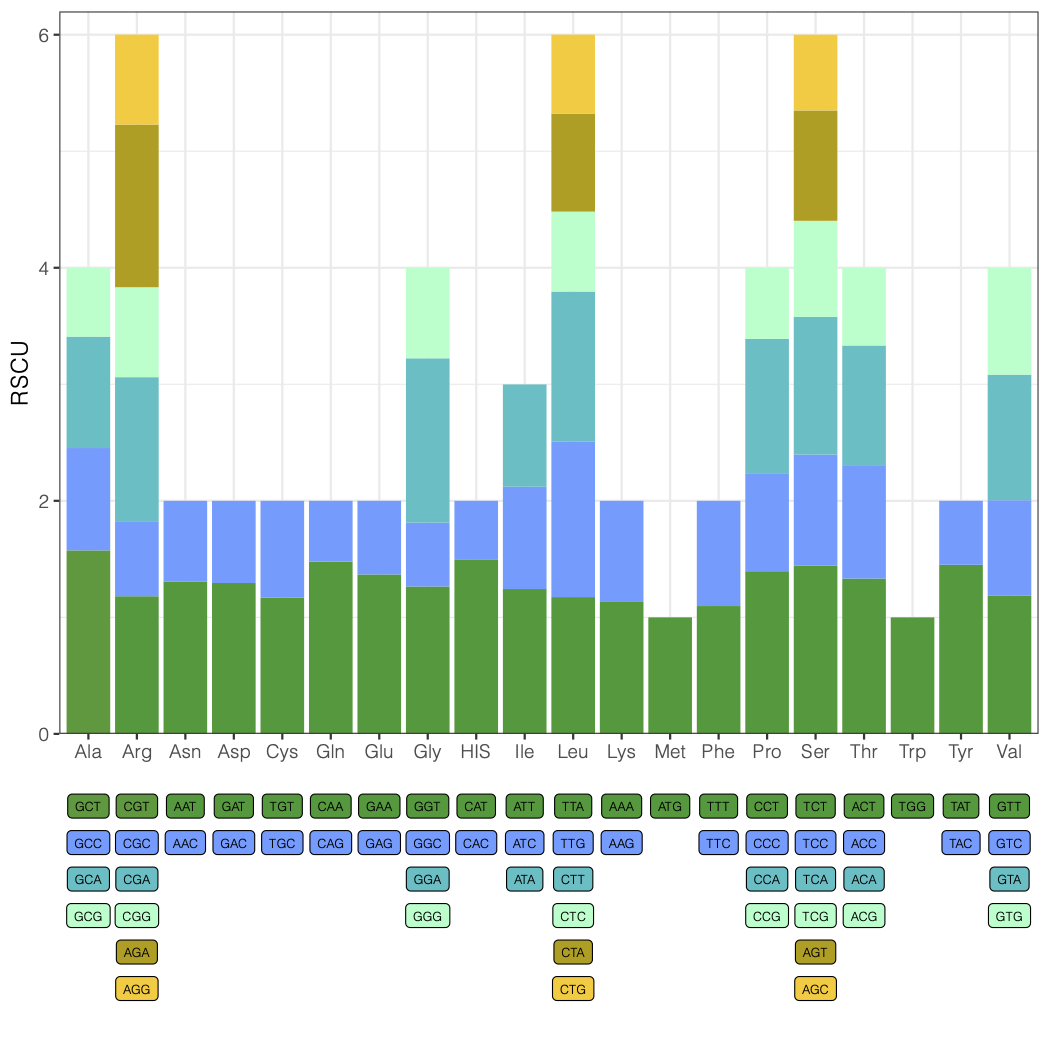


**Fig. S1** Relative synonymous codon usage (RSCU) in PCGs of *D. hancockii* mitogenome.

Codon families are on the abscissa.
