# Supplementary material for: Mitochondrial genome insights into the spatio-temporal distribution and genetic diversity of Dendrobium hancockii Rolfe (Orchidaceae)
Source: Front Plant Sci. 2024 Oct 22;15:1469267. doi: 10.3389/fpls.2024.1469267 (PMC11535511; doi:10.3389/fpls.2024.1469267)
Supplement: Supplementary file 1 [file DataSheet1.zip › Supplementary Materials/Figure_S2_new.docx]

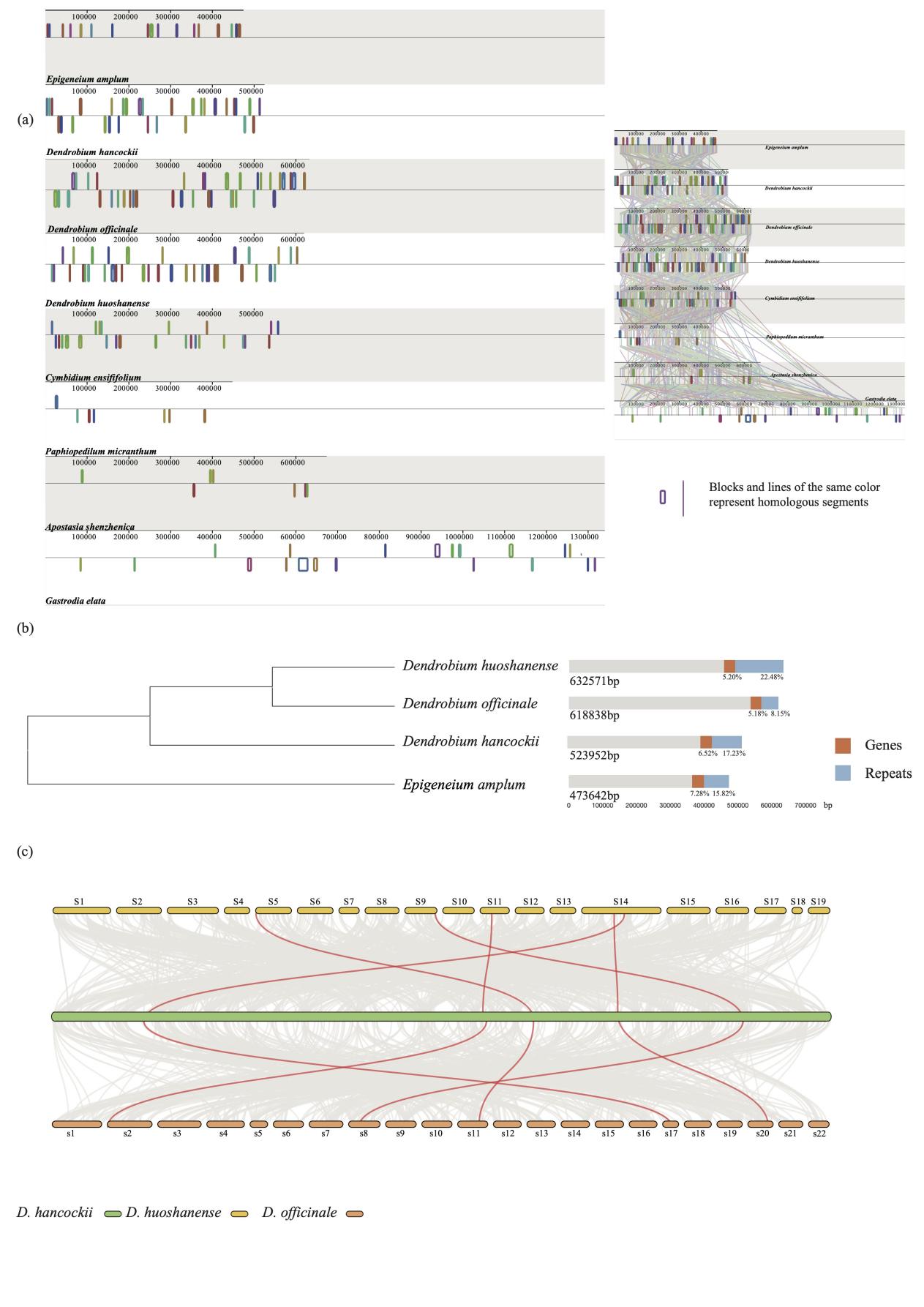


**Fig. S2** Mitochondrial Comparative Genome Analysis of Orchidaceae Species:

1. Distribution of homologous blocks of eight Orchidaceae plants analyzed by Mauve. (b) Evolutionary relationships within the *Dendrobium* genus, along with the distribution proportions of the genome and repetitive sequences. (c) Collinearity analysis, with red regions representing five identified conserved gene clusters.
