# Supplementary material for: Mitochondrial genome insights into the spatio-temporal distribution and genetic diversity of Dendrobium hancockii Rolfe (Orchidaceae)
Source: Front Plant Sci. 2024 Oct 22;15:1469267. doi: 10.3389/fpls.2024.1469267 (PMC11535511; doi:10.3389/fpls.2024.1469267)
Supplement: Supplementary file 1 [file DataSheet1.zip › Supplementary Materials/Table S1 Primers used for PCR.docx]

**Table S1. Primers used for PCR**

| **Primer name** | **Forward primer (5’to 3’)** | **Reverse primer (5’to 3’)** | **Tm (°C)** |
| --- | --- | --- | --- |
| Primer for *Dhan001870* | CATCATATAAAGTGCATTACATGGTGG | GGCTTCAAAAATTAAAATTGGGACCAT | 57 |
| Primer 1 for *Dhan001925* | GCATGATTGGGAGATCTAATTGGCA | CCATAGTTCAAGACCTCCTTCCC | 58 |
| Primer 2 for *Dhan001925* | GTGACTACTGCATTGCTGAATCAC | CTATTTCACCATCACTCTGAACAATAGAC | 58 |
| Primer for *Dhan002197* | GCTCCACTGTAGGATCTATCAGCT | CTTTTCTCCTGCAATCAGAACTAAGG | 59 |
| Primer for *Dhan009708* | CTATTGTTCTAATGCGGCAGCAAT | GCTTTAAGATTAACGGCCTCCCTC | 59 |
| Primer for *Dhan020148* | CCTCTCTTATTTAGGCAACCAGCT | GGATGATGAAAATCAGCACCAGAG | 58 |
| Primer for *Dhan021861* | GTGGTGACATATACTTCGGTAATTAG | CTTTTTGGAAGAAGGCCATCTTTTG | 57 |
| Primer 1 for *Dhan023317* | CTTGTTGCTTGAAAGAAAGCTCTT | CGTGTTCTTTGCAAAAACAAGAATGT | 59 |
| Primer 2 for *Dhan023317* | CTCATTCCAGCGTCATTATTGATAA | CTTCGTAGCCCACTATGCTTC | 57 |
| Primer 1 for *Dhan003705* | CAATCAAATTCAGGTCTTGAGAACACT | CAGTTCTTGCCTAATGTGCTTATATTTG | 58 |
| Primer 2 for *Dhan003705* | CATGATTTTGCACTATCCATGGCT | CTCCTCCTCCTTTCTTTCCCGATA | 58 |
