# Supplementary material for: Mitochondrial genome insights into the spatio-temporal distribution and genetic diversity of Dendrobium hancockii Rolfe (Orchidaceae)
Source: Front Plant Sci. 2024 Oct 22;15:1469267. doi: 10.3389/fpls.2024.1469267 (PMC11535511; doi:10.3389/fpls.2024.1469267)
Supplement: Supplementary file 1 [file DataSheet1.zip › Supplementary Materials/Table S2 Mitochondrial Reference Genome Database.docx]

**Table S2. Accession numbers of the Mitochondrial Reference Genome Database**

| **Taxonomy** | **Species** | **GenBank Accession No.** |
| --- | --- | --- |
| Apiales | *Bupleurum falcatum* | KX887330.1 |
| Asterales | *Codonopsis lanceolata* | MG775430.1 |
|  | *Lactuca serriola* | NC_042378.1 |
| Asparagales | *Gastrodia elata* | MF070084.1-MF070102.1 |
|  | *Allium cepa* | NC_030100.1 |
|  | *Chlorophytum comosum* | MW411187.1 |
|  | *Crocus sativus* | OL804177.1 |
|  | *Hemerocallis citrina* | MZ726801.1-MZ726803.1 |
|  | *Dendrobium huoshanense* | LC657527‐LC657545 |
|  | *Dendrobium officinale* | LC640134‐LC640155 |
| Brassicales | *Brassica juncea* | NC_016123.1 |
|  | *Carica papaya* | EU431224.1 |
|  | *Eruca vesicaria* | KF442616.1 |
|  | *Raphanus sativus* | AP018472.1 |
|  | *Turritis glabra* | LC325489.1 |
|  | *Arabidopsis thaliana* | NC_037304.1 |
| Caryophyllales | *Beta vulgaris* | BA000009.1 |
| Cucurbitales | *Citrullus lanatus* | GQ856147.1 |
|  | *Cucurbita pepo* | GQ856148.1 |
| Fabales | *Acacia ligulata* | NC_040998.1 |
|  | *Ammopiptanthus mongolicus* | NC_039660.1 |
|  | *Glycine soja* | NC_039768.1 |
| Magnoliales | *Liriodendron tulipifera* | MK340747.1 |
| Malpighiales | *Hevea brasiliensis* | AP014526.1 |
|  | *Ricinus communis* | NC_015141.1 |
|  | *Salix sinopurpurea* | NC_029693.1 |
| Malvales | *Bombax ceiba* | NC_038052.1 |
|  | *Gossypium barbadense* | NC_028254.1 |
| [Poales](https://www.iplant.cn/info/Poales) | *Oryza sativa cultivar Shen95* | NC_066488.1 |
|  | *Zea mays subsp. parviglumis* | NC_008332.1 |
| Ranunculales | *Nymphaea colorata* | NC_037468.1 |
| Rosales | *Cannabis sativa* | NC_029855.1 |
|  | *Malus hupehensis* | KR534606.1 |
|  | *Ziziphus jujuba* | NC_029809.1 |
| Solanales | *Capsicum annuum* | KJ865410.1 |
| Vitales | *Vitis vinifera* | FM179380.1 |
